# Evaluation of an Intranasal Virosomal Vaccine against Respiratory Syncytial Virus in Mice: Effect of TLR2 and NOD2 Ligands on Induction of Systemic and Mucosal Immune Responses

**DOI:** 10.1371/journal.pone.0061287

**Published:** 2013-04-08

**Authors:** Muhammad Shafique, Tjarko Meijerhof, Jan Wilschut, Aalzen de Haan

**Affiliations:** Department of Medical Microbiology, Molecular Virology Section, University Medical Center Groningen and University of Groningen, Groningen, The Netherlands; The Ohio State University, United States of America

## Abstract

**Introduction:**

RSV infection remains a serious threat to newborns and the elderly. Currently, there is no vaccine available to prevent RSV infection. A mucosal RSV vaccine would be attractive as it could induce mucosal as well as systemic antibodies, capable of protecting both the upper and lower respiratory tract. Previously, we reported on a virosomal RSV vaccine for intramuscular injection with intrinsic adjuvant properties mediated by an incorporated lipophilic Toll-like receptor 2 (TLR2) ligand. However, it has not been investigated whether this virosomal RSV vaccine candidate would be suitable for use in mucosal immunization strategies and if additional incorporation of other innate receptor ligands, like NOD2-ligand, could further enhance the immunogenicity and protective efficacy of the vaccine.

**Objective:**

To explore if intranasal (IN) immunization with a virosomal RSV vaccine, supplemented with TLR2 and/or NOD2-ligands, is an effective strategy to induce RSV-specific immunity.

**Methods:**

We produced RSV-virosomes carrying TLR2 (Pam_3_CSK_4_) and/or NOD2 (L18-MDP) ligands. We tested the immunopotentiating properties of these virosomes *in vitro*, using TLR2- and/or NOD2-ligand-responsive murine and human cell lines, and *in vivo* by assessing induction of protective antibody and cellular responses upon IN immunization of BALB/c mice.

**Results:**

Incorporation of Pam_3_CSK_4_ and/or L18-MDP potentiates the capacity of virosomes to activate (antigen-presenting) cells *in vitro*, as demonstrated by NF-κB induction. *In vivo*, incorporation of Pam_3_CSK_4_ in virosomes boosted serum IgG antibody responses and mucosal antibody responses after IN immunization. While L18-MDP alone was ineffective, incorporation of L18-MDP in Pam_3_CSK_4_-carrying virosomes further boosted mucosal antibody responses. Finally, IN immunization with adjuvanted virosomes, particularly Pam_3_CSK_4_/L18-MDP-adjuvanted-virosomes, protected mice against infection with RSV, without priming for enhanced disease.

**Conclusion:**

Mucosal immunization with RSV-virosomes, supplemented with incorporated TLR2- and/or NOD2-ligands, represents a promising approach to induce effective and safe RSV-specific immunity.

## Introduction

Respiratory Syncytial Virus (RSV) is the major cause of lower respiratory tract infections, particularly in infants and children. According to the WHO reports, RSV causes 64 million infections annually, leading to the hospitalization of 18,000–75,000 children in the USA alone with an estimated mortality of 160,000 [1]. Most children are infected at least once by the age of 2 and reinfection may occur throughout life due to incomplete immunity to RSV [2], [3]. RSV, therefore, remains a threat at older age, particularly in risk groups such as the elderly and immuno-compromised individuals. Despite the burden of RSV disease, there is still no licensed vaccine against RSV infection.

New candidate non-replicating RSV vaccines should induce protective immunity without priming for enhanced respiratory disease (ERD) upon natural infection, as did the formalin-inactivated and alum-adjuvanted whole RSV vaccine (FI-RSV) used in a clinical trial in the 1960s [4]. Possible factors involved in priming for ERD by non-replicating vaccines include disruption of protective epitopes by the chemical inactivation of the virus, poor innate receptor activation by the vaccine resulting in induction of poorly neutralizing antibodies and excess Th2-type responses [5], [6]. Recent studies indicate that addition of Toll-like receptor (TLR) ligands, used as vaccine adjuvants, improve antibody affinity and Th1-skewing and prevent priming for ERD [7], [8]. Furthermore, mucosal (i.e., intranasal, IN) immunization prevents induction of this complication and additionally induces secretory IgA (S-IgA) responses in the respiratory tract, which can act as a first line of defense against RSV [8], [9]. Thus, new candidate RSV vaccines should induce Th1-skewed immune responses with induction of protective systemic and mucosal antibodies without priming for enhanced pathology upon natural infection. Mucosal vaccines that include TLR-ligands for activation of innate receptors could be promising in this respect.

Virosomes are non-replicating virus-like particles consisting of reconstituted membranes of enveloped viruses [10]. The production of virosomes does not use chemicals (e.g. formalin) that could possibly modify protective epitopes. Upon production, virosomes allow the incorporation of lipophilic adjuvants, such as lipophilic TLR-or NOD-like receptor (NLR)ligands, in their membranes. We previously reported on the feasibility of inclusion of lipophilic TLR-ligand adjuvants (i.e. TLR2-ligand Pam_3_CSK_4_ and TLR4-ligand Monophosphoryl Lipid A; MPLA) in RSV virosomes and demonstrated that such adjuvant-supplemented virosomes have the capacity to induce protective antibodies after parenteral administration to mice or cotton rats, without priming for enhanced disease [11], [12]. However, we have not yet investigated whether such virosomal RSV vaccine candidates are suited for use in mucosal immunization strategies.

TLR ligands have been reported to have the capacity to potentiate immune responses against mucosally delivered antigens[13]. In this respect, we found that a TLR9 ligand (i.e. CpG DNA), alone or co-formulated with a NOD2 ligand (i.e. L18-MDP), could boost mucosal and systemic antibody responses to admixed inactivated RSV whole virions upon IN administration to mice [8]. However, lipophilic TLR ligands like Pam_3_CSK_4_ or MPLA can be much more efficiently incorporated in virosomes compared to CpG DNA [11], [12]. The latter also would need additional incorporation of cationic lipids in the virosomal membrane in order to bind the negatively charged DNA molecules [14]. We therefore chose to explore the use of Pam_3_CSK_4_ as a TLR ligand adjuvant in virosomes. The TLR for Pam_3_CSK_4_, i.e. TLR2, is abundantly expressed on many cell types in mucosal tissues and does not need additional co-receptors like those described for the receptor of MPLA (TLR4), i.e. CD14 and MD2, that have low expression levels in mucosal tissues [15]. Also, other TLR2 ligands have shown promise as mucosal adjuvants [16]–[18].

In this study, we explored the use of the TLR2 ligand Pam_3_CSK_4_ in RSV-virosomes for potentiation of immune responses. We further investigated the use of a NOD2 ligand with possible mucosal immuno-adjuvant properties, i.e. L18-MDP [19], [20], and its combined use with the TLR2 ligand, Pam_3_CSK_4_. The rationale for the combined use is that NOD2 ligands may synergistically enhance immune activation induced by TLR-ligands [21]–[23], which would result in a better immunopotentiation by the mucosal virosomal RSV-vaccine.We demonstrate that incorporation of TLR2 ligands and/or NOD2 ligands in virosomes potentiates their capacity to activate a mouse macrophage cell line and human TLR/NOD2-expressing cells *in vitro*. *In vivo*, incorporation of a TLR2-ligand in virosomes boosted RSV-specific serum IgG and mucosal IgA responses after IN immunization of mice. While virosome incorporation of NOD2-ligand alone did not potentiate antibody responses, incorporation of NOD2 ligand in virosomes carrying a TLR2 ligand further stimulated local IgA and serum IgG responses. Adjuvantation of RSV virosomes with TLR2/NOD2 ligands also primed for a Th1-skewed response. Finally, RSV virosomes adjuvanted with TLR2/NOD2 ligands protected mice against challenge with infectious RSV without inducing enhanced disease.

## Materials and Methods

### Ethics statement

All animal experiments were evaluated and approved by the Committee for Animal Experimentation (DEC) of the University Medical Center Groningen, University of Groningen, The Netherlands, according to the guidelines provided by the Dutch Animal Protection Act (permit number DEC 5239B). Immunizations and challenges were carried out under isoflurane anesthesia and every possible effort was made to minimize suffering of the animals.

### Virus production and cell culture

RSV strain A2 (ATCC VR 1540) was kindly donated by Mymetics BV (Leiden, The Netherlands). The virus was grown in roller bottles on HEp-2 cells (ATCC, CL-23, Wesel, Germany) in HEp-2 medium: DMEM (Invitrogen, Breda, The Netherlands) supplemented with Pen/Strep, L-Glutamine, Sodium bicarbonate, HEPES, Sodium pyruvate, 1× non-essential amino acids (all from Invitrogen) and 10% FBS (Lonza-Biowhittkar, Basel, Switzerland), and purified by a combination of differential and rate zonal ultracentrifugation on sucrose gradients. Purified virus was snap-frozen in liquid nitrogen and stored at −80°C in 20% sucrose in HNE buffer (5 mM Hepes, 145 mM NaCl, 1 mM EDTA, pH 7.4).

RAW-Blue (Mouse Macrophage Reporter Cell Line), HEK-Blue TLR2, HEK-Blue Null1, HEK-Blue NOD2, HEK-Blue Null2 cell lines, were purchased from Invivogen (Toulouse, France) and maintained according to the manufacturer's instructions. The abbreviation HEK stands for Human Embryonic Kidney.

### Preparation of vaccine formulations

Virosomal RSV vaccine was produced as described earlier [11]. Briefly, purified virus was pelleted by ultracentrifugation for 30 min at 40, 000 rpm at 4°C, and the pellets were suspended in sterile HNE buffer. Then, this suspension was mixed with an equal volume of 200 mM 1,2-dihexanoyl-*sn*-glycero-3-phosphocholine (DCPC) resulting in dissolution of the viral envelopes. The viral nucleocapsid was removed by ultracentrifugation at 50,000 rpm for 30 min at 4°C. Then, the supernatant containing the viral envelopes was added to a thin film of lipids prepared in a glass tube of 2∶1 molar mixture of egg phosphatidylcholine (PC) and egg phosphatidylethanolamine (PE) (Avanti Polar Lipids, Alabaster, AL, USA) in 2∶1 chloroform/methanol at 850 nmol/mg of viral envelop proteins. The lipid mixture was evaporated to dryness on the wall of a glass tube and traces of the solvents were removed at a high vacuum. The lipopeptide adjuvant, N-pamitoyl-S-[2,3-bis(palmitoyloxy)-(*2RS*)-propyl]-[*R*]-cysteinyl-[*S*]-seryl-[*S*]-(lysyl)3-lysine (Pam_3_CSK_4_,EMC Microcollections GmbH, Tubingen, Germany, lyophilized from the HCl solution), and/or L-18 muramyldipeptide (L18-MDP) (6-*O-*stearoyl-N-Acetyl-muramyl-L-alanyl-D-isoglutamine; Invivogen, Toulouse, France) were dissolved in 100 mM DCPC in HNE, pH 7.4 and the solution was filtered through a 0.22 µm filter. To prepare virosomes, supernatant containing the viral envelopes and DCPC was combined with a thin film of lipid mixture, while to prepare adjuvanted virosomes, the lipopeptide solutions (Pam_3_CSK_4_) and/or L18-MDP were added separately or together (1 mg of adjuvant(s) per mg of viral protein). The mixture was incubated for 15 min at 4°C, filtered through 0.22 µm filter and dialyzed against 4×2 liters of HNE buffer pH 7.4 in a sterile slide-A-lyzer (10 kD cut-off; ThermoScientific, Etten, Leur, the Netherlands) for 48 h. The buffer was changed 4 times. The virosomes were harvested and protein concentration was determined by Bio-Rad Bradford protein assay.

FI-RSV vaccine was prepared according to the protocol, which was used for the 1960s FI-RSV vaccine preparation as reported in [24]. This vaccine was diluted in HNE buffer to contain 5 µg of RSV protein in 25 µl.

### In vitro analyses

The virosomal formulations were analyzed by equilibrium density gradient centrifugation on 10–60% sucrose gradients in HNE. The gradients were centrifuged for 60 h in an SW55 Ti rotor at 50000 rpm and the samples from the gradients were analyzed for protein, phospholipid and density (by refractometry). Later, each fraction was dialyzed against HNE in a slide-A-Lyzer MINI Dialysis device (Thermo Scientific, Geel, Belgium) overnight to remove the sucrose. Then, samples were corrected for increase in volume due to dialysis and 100 µl of the samples were used to stimulate each cell line i.e. Mouse Macrophage Reporter Cell Line (RAW-Blue cells) and Human Embryonic Kidney cell lines (HEK-Blue TLR2, HEK-Blue Null1, HEK-Blue NOD2, HEK-Blue Null2). RAW-Blue cells were used to measure vaccine/innate receptor ligand-induced NF-κB activation. These cells express all TLRs (except TLR5) as well as RIG-I, MDA5, NOD1 and NOD2 and carry a NF-κB responsive-gene encoding secreted alkaline phosphatase. RAW-Blue Cells (1×10^5^ cells/well in 100 µl) were incubated with 100 µl sample overnight at 37°C in a 96-well flat bottom plates in triplicate. Alkaline phosphatase was quantified by incubating 20 µl cell supernatants with 180 µl Quanti-Blue (Invivogen, Toulouse, France) for 30 min at 37°C. Next, absorbance was measured at 630 nm through plate reader. Next, the relative amount of NF-κB induced by the gradient (virosomal) fractions was calculated by comparing to the NF-κB induced by CpG ODN, which was used as positive control. To study the stimulating capacity of the (virosomal) fractions to activate human TLR, HEK-Blue cells (HEK-TLR2, HEK-Null1, HEK-NOD2 and HEK-Null2; 5×10^4^ cells/well) were incubated with 100 µl of the (virosomal) fractions in a 96-well flat bottom plate overnight at 37°C, 5% CO_2_ atmosphere. Secreted alkaline phosphatase was assayed as indicated above. The relative amount of NF-κB induced in TLR2/Null1 and NOD2/Null2 cells was calculated by comparing to NF-κB induced by TNFα (100 ng/ml) stimulation, used as positive control.

### Immunization schedule and RSV challenge

Female specified-pathogen-free BALB/c OlaHsd mice (6–8 weeks old) purchased from Harlan, Zeist, The Netherlands, were used for all immunization experiments. Mice were immunized either with RSV virosomes (5 µg) alone or with incorporated innate receptor ligands, i.e. TLR2 (Pam_3_CsK_4_) and/or NOD2-ligands (L18-MDP) present at a 1∶1 weight ratio of ligand to vaccine antigen, respectively. Mice (6 mice per group) were immunized on days 0 and 21, under 3–4.5% isoflurane anesthesia in O_2_ by IN inoculation of 50 µl. One group of mice was immunized with FI-RSV vaccine by intramuscular (IM) injection of 25 µl of FI-RSV absorbed to aluminium hydroxide (see above) and served as a control for vaccine-induced ERD. Another group of mice was immunized by IN inoculation with live-virus (1×10^6^ TCID_50_) and served as a control for optimal anti-viral immunity. On day 28, all mice were challenged with live-virus (1×10^6^ TCID_50_) by administration of 5×10 µl of virus in the nose under isoflurane anesthesia.

### Collection of blood samples and mucosal washes

Blood samples were drawn twice during the experiment: on day 28 before challenge by orbital puncture and day 32 by heart puncture. Sera were obtained after centrifugation of coagulated blood at 12,000 rpm for 10 min, and samples were stored at −20°C until further analysis. Bronchoalveolar lavages (BAL) and nasal washes were performed as previously described [25]. Briefly, lung lavages were performed by gentle injection of 1 ml PBS into the lungs with a syringe connected to the trachea, followed by subsequent aspiration of 1 ml of the wash fluid. Nasal washes were done by injection of 1 ml PBS retrograde via the trachea into the naso-pharynx and the lavage fluid was collected at the nostrils. The cellular components in the washes were removed by low-speed centrifugation. The supernatants were stored at −20°C until further analysis.

### Antibody titer determination through ELISA

The antibody response to RSV was determined using enzyme-linked immunosorbent assay (ELISA). ELISA plates (Greiner Bio-one, Alphen a/d Rijn, The Netherlands) were coated with beta-propiolactone (BPL) inactivated whole RSV (BPL-RSV) at 0.5 µg protein per well in coating buffer (0.05 M carbonate–bicarbonate, pH 9.6–9.8) overnight at 37°C. Plates were washed three times with coating buffer and blocked with a 2.5% solution of milk powder (Protifar Plus, Nutricia, Zoetermeer, The Netherlands) in coating buffer for 45 min at 37°C, then washed twice with coating buffer and three times with PBS Tween (PBST), containing 0.05% Tween 20 (Merck, Schiphol-Rijk, The Netherlands). Serial two-fold dilutions of serum samples (for IgG, IgG1 and Ig2a, IgA, IgE) and BAL and nasal wash samples (for IgA, IgG determination) were applied to the plates and incubated for 90 min. Plates were washed three times with PBST and incubated with a 1∶5000 dilution of horseradish-peroxidase conjugated goat anti-mouse IgG, IgG1, IgG2a or IgA; Southern Biotech, Birmingham, AL, USA) for 60 min at 37°C. Subsequently, the plates were washed three times with PBST and three times with PBS. After aspiration, *O*-Phenylenediamine (OPD; Sigma-Aldrich, St Louis, MO, USA) in 50 mM phosphate buffer pH 5.6 with 0.02% H_2_O_2_ was added and wells were incubated for 30 min. Then, the reaction was stopped by adding 50 µl 2 M H_2_SO_4_ per well and the optical densities (OD) of the wells at 490 nm was determined. IgA levels were expressed as OD-values of undiluted samples. IgG levels were expressed as titers and defined as the reciprocal of the highest dilution that gave an OD value of at least 0.2.

### IFN-γ and IL-5 detection in stimulated splenocyte supernatants

Four days after the virus challenge, mice were sacrificed and spleens were harvested separately in 15 ml tubes containing Iscove's Modified Dulbecco's Medium (IMDM; Invitrogen, Breda, The Netherlands) supplemented with 1% Penicillin/Streptomycin and 0.1% beta-mercaptoethanol (Invitrogen, Breda, The Netherlands) and 10% FCS (Lonza-Biowhittaker, Basel, Switzerland). Then, spleens were processed individually for *in vitro* stimulation. Briefly, washed spleens were passed through a 70 µm mesh (BD Biosciences, Heidelberg, Germany) using sterile 3 ml syringe plungers. Subsequently, erythrocytes were lysed by incubating with hypotonic medium (0.83% NH_4_Cl, 10 mM KHCO_3_, 0.1 mM EDTA, pH 7.2) for 5 min on ice. The cells were washed with IMDM, counted and brought to appropriate concentrations. Fresh spleen cells were seeded into 96-well plates at a concentration of 2×10^6^ cells/ml and stimulated with BPL-RSV (10 µg/ml) in IMDM/10% FCS in triplicates and incubated at 37°C in a 5% CO_2_ atmosphere for 72 hrs. Supernatants were harvested and stored at −20°C until further analysis. IFN-γ and IL-5 cytokines were measured in supernatants of these stimulated splenocytes. For this, mouse IFN-γ- and mouse IL-5- high sensitivity ELISA kits (eBioscience, Vienna, Austria) were used according to the manufacturer's instruction. Detection limits were 15 pg/ml and 4 pg/ml for IFN-γ and IL-5, respectively.

### Lung virus titration

Lungs were removed aseptically from all mice following euthanasia and washed in Dulbecco's Modified Essential Medium (DMEM), (PAA Laboratories, Colbe, Germany), supplemented with 2% FCS and transferred into 4 ml tubes containing 1 ml medium. Then, the lungs were homogenized individually with an automated Potter homogenizer Polytron-Aggregate® (Thomas Scientific, Swedesboro, NJ, USA), centrifuged at 1400 rpm for 10 min at 4°C and supernatants were separated. Virus titers were determined, by titration of the tissue-culture infectious dose (TCID_50_). Briefly, a serial two fold dilutions of these samples were made in 96-well plates in quadruplicates with 1∶5 starting dilution. Hep-2 cells, (20,000 per well) were seeded to the virus dilutions and incubated for 5 days at 37°C in a 5% CO_2_ atmosphere. Then, supernatants were removed and plates were washed with PBS. The cells were then fixed with 1% para-formaldehyde in PBS for 1 h. After blocking cells with 2% milk powder (Protifar plus, Nutricia, Zoetermeer, The Netherlands) in PBS for 45 min at 37°C, plates were stained with 50 µl 1∶400 dilution of FITC-labeled goat anti-RSV antibody (Meridian life science Inc, Saco, ME, USA) at 37°C overnight. The next day, plates were washed with PBS and analyzed under fluorescent microscope. Wells were considered positive for infection when ≥1 fluorescent syncytium was detected. Finally, TCID_50_ titers were calculated by the Reed-Muench method using an Excel spreadsheet.

### Lung Histopathology

The lung lobes were harvested four days post infection, inflated with 4 % formalin in PBS for overnight and subsequently embedded in paraffin. Then, four µm slices were prepared, stained with standard hematoxylin and eosin (H & E) and were photographed using Nanozomer (Hamamatsu). Each lung section was analyzed for one of the following four parameters of pulmonary inflammatory changes: peribronchiolitis (inflammatory cells surrounding a bronchiole), perivasculitis (inflammatory cells surrounding a small blood vessel), alveolitis (inflammatory cells within alveolar spaces), and interstitial pneumonitis (increased thickness of alveolar walls associated with inflammatory cells) by light microscopic analysis of slides.

### Data analysis

All statistical analyses were performed using Graphpad Prism v5.0 (Graphpad Software, San Diego California, USA). Statistical significance was determined using unpaired Mann-Whitney U test. P values ≤ 0.05 were considered statistically significant.

## Results

### Characterization of virosomal formulations

**Figure 1:**
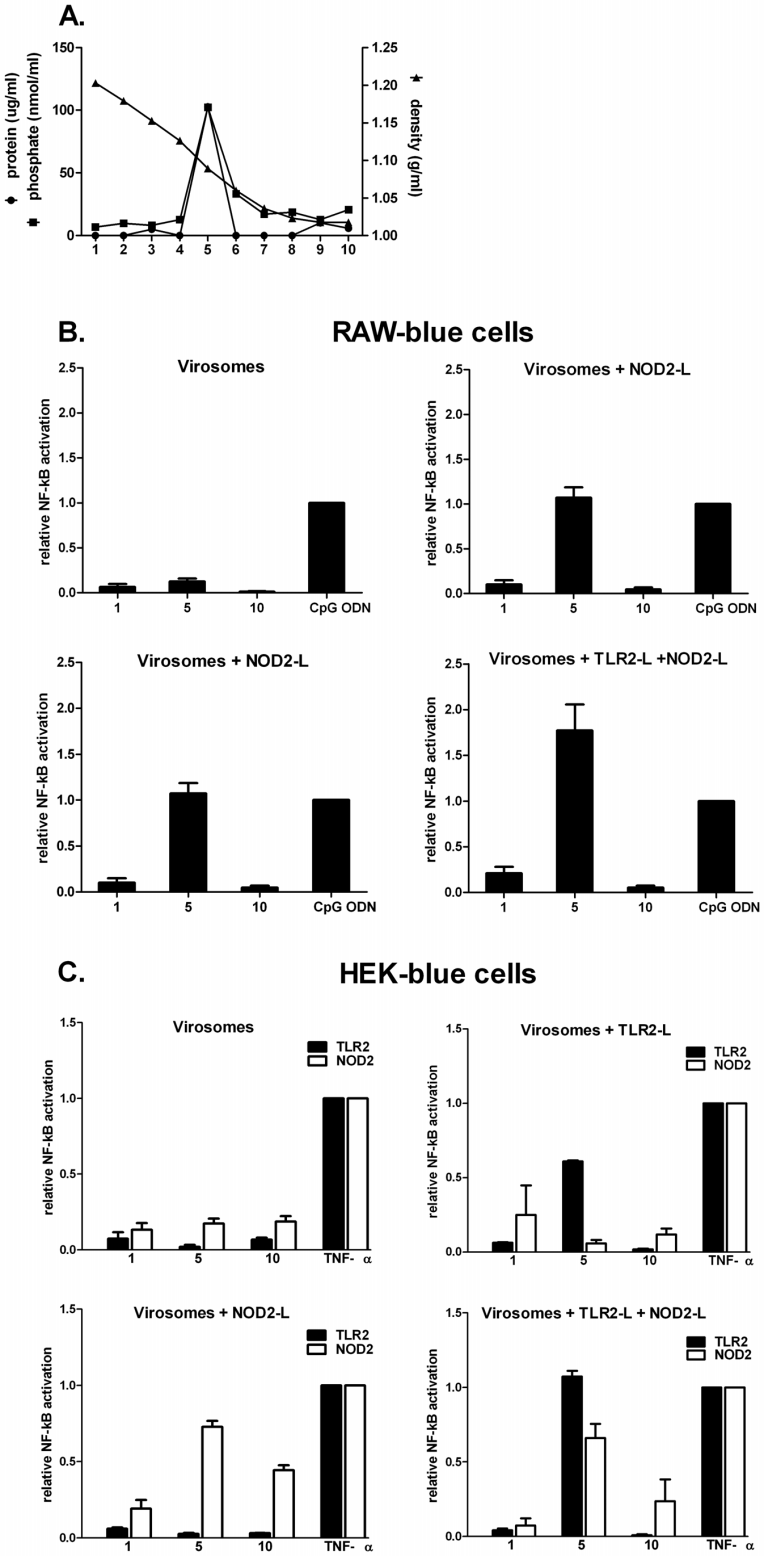
*In vitro* analysis of RSV virosomes and RSV virosomes adjuvanted with TLR2 and/or NOD2ligands. RSV virosomes and RSV virosomes adjuvanted with TLR2 and/or NOD2 ligands were spun on an equilibrium density sucrose gradient. Subsequently, density, phospholipids and protein concentrations of each fraction was determined. Panel A shows a representative profile of a virosome purification gradient. Fractions (1,5,10; representing bottom, virosomal and top gradient fractions, respectively) were analyzed to determine their capacity to activate NF-κB in mouse macrophages (RAW-Blue cells; panel B) and human embryonic kidney cells (HEK-BlueTLR2 & HEK-Blue NOD2 cells; panel C). The level of NF-κB induced in RAW-blue cells was expressed as values relative to levels of NF-κB induced by CpG ODN, the positive control. To assess non-specific NF-κB activation by TLR2 and NOD2 ligand-carrying virosomes in HEK cells, control cells (HEK-Blue Null1 & HEK-Blue Null2 cells, respectively) were incubated with the same fractions and these values were subtracted from values obtained with HEK-BlueTLR2 & HEK-Blue NOD2 cells, respectively. As a control, HEK-Blue TLR2 and HEK-Blue NOD2 cells were stimulated with 100 ng/ml TNF-α. Bars represent the NF-κB activation relative to TNF-α control.

Virosomal RSV formulations were prepared according to the protocol described in the Materials and Methods section. For all virosomal RSV-preparations, protein and phospholipids were found to co-migrate in the density gradients, indicating the successful formation of virosomes (Figure 1A). To investigate whether the lipophilic adjuvants were associated with the RSV virosomes, gradient fractions containing the virosomes, and top and bottom gradient fractions without virosomes (as controls), were tested for their capacity to induce NF-κB in TLR or NOD2 receptor-expressing mouse macrophage cell lines *in vitro*. Non-adjuvanted RSV virosome fractions poorly induced NF-κB expression in this assay (Figure 1B). Incorporation of Pam_3_CSK_4_ or L18-MDP in RSV virosomes clearly potentiated the capacity of the virosomes to induce NF-κB (Figure 1B). Incorporation of both ligands in virosomes enhanced NF-κB induction compared to the NF-κB induction by single incorporated ligands, although not in a synergistic fashion (Figure 1B). Top and bottom gradient fractions induced low NF-κB levels, suggesting that most of the added ligands was efficiently incorporated in the virosomal membranes.

Virosomal RSV formulations were also added to human cell lines that express single human innate receptors. Non-adjuvanted RSV virosomes again poorly induced NF-κB expression in TLR2 or NOD2-expressing cell lines (i.e. HEK-TLR2- or HEK-NOD2) cells, respectively (Figure 1C). RSV virosomes with incorporated Pam_3_CSK_4_ enhanced NF-κB expression in HEK-TLR2 cells, but not in HEK-NOD2 cells. Similarly, RSV virosomes with incorporated L18-MDP enhanced NF-κB expression in HEK-NOD2 cells, but not in HEK-TLR2 cells. Some residual bioactivity of L18-MDP was seen in the top fraction from the RSV-L18-MDP virosome density gradient, suggesting that most, but not all ligand was incorporated into the viral membranes. Since a large proportion of the lipophilic ligands was found to be incorporated into the viral membranes, we used non-fractionated virosomes for all subsequent immunization experiments.

### *In vivo* immunogenicity

To evaluate the immunogenicity of the virosomal preparations upon mucosal administration, mice were immunized intranasally (IN) with RSV virosomes alone or RSV virosomes with incorporated Pam_3_CSK_4_ and/or L18-MDP. Control groups included non-immunized mice (HNE group), and mice immunized by live virus infection. One group of mice was immunized IM with FI-RSV vaccine to represent the mirror image of the FI-RSV vaccine used in 1960s clinical trial mentioned above.

**Figure 2:**
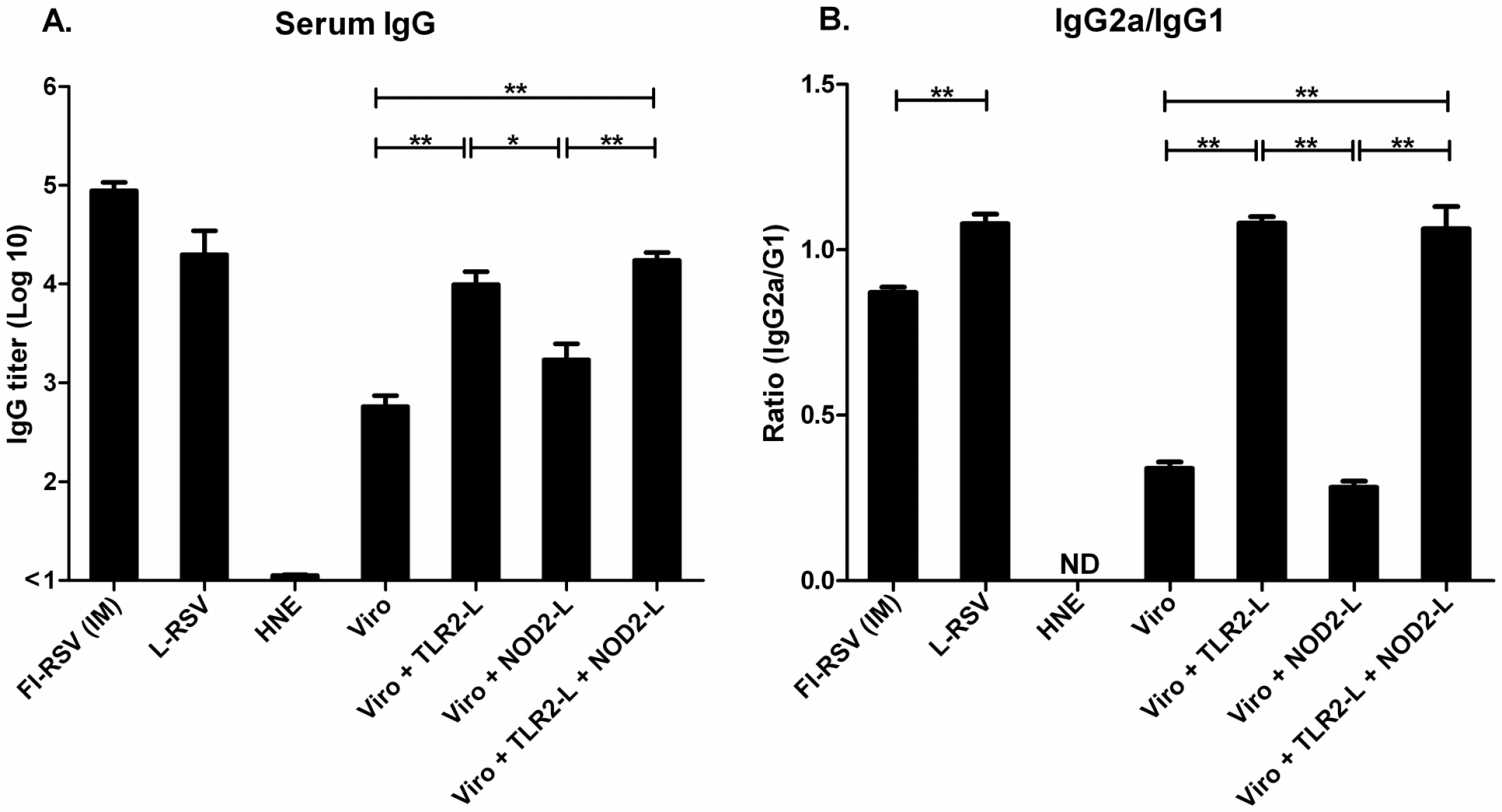
RSV-specific systemic IgG antibody responses after IN immunization of mice. BALB/c mice were immunized IN with RSV virosomal vaccine formulations (5 µg of protein) or HNE. Control mouse groups were either immunized IM with FI-RSV or IN with live-RSV (L-RSV) on day 0 and 21. Six mice were used in each group. One week after the booster immunization, RSV-specific IgG responses in serum (A) and IgG-subtypes (IgG2a/IgG1) (B) were determined by ELISA. Panel A: Bars represent the geometric mean titer and standard deviation. Panel B: Bars represent the ratios of IgG2a/IgG1. The data shown are representative of at least 3 separate experiments. Data was analyzed by a Mann-Whitney U test and a p-value of≤0.05 was considered to represent a significant difference. * p≤0.05, ** p≤0.01.

Mice immunized with RSV virosomes with incorporated Pam_3_CSK_4_ showed significantly higher IgG antibody responses compared to responses induced by non-adjuvanted RSV virosomes (Figure 2A). Incorporation of L18-MDP in virosomes also induced increased IgG antibody responses. Moreover, additional incorporation of L18-MDP into the Pam_3_CSK_4_-containing virosomes further enhanced IgG antibody responses.

In order to analyze the phenotype of the immune responses, Th1-signature IgG2a and Th2-signature IgG1 subtype antibodies were determined. Incorporation of Pam_3_CSK_4_ alone or combined with L18-MDP into RSV virosomes resulted in a significant increase of IgG2a/IgG1 ratios after IN-immunization of mice (Figure 2B). Incorporation of L18-MDP alone did not result in an increase of IgG2a/IgG1 ratios. As expected, live RSV induced higher IgG2a/IgG1 ratios than FI-RSV (Figure 2B). Thus, incorporation of TLR2/NOD2 ligands in IN-administered RSV virosomes significantly stimulates systemic RSV-specific IgG antibody responses with a more pronounced production of Th1-signature IgG2a antibodies.

**Figure 3:**
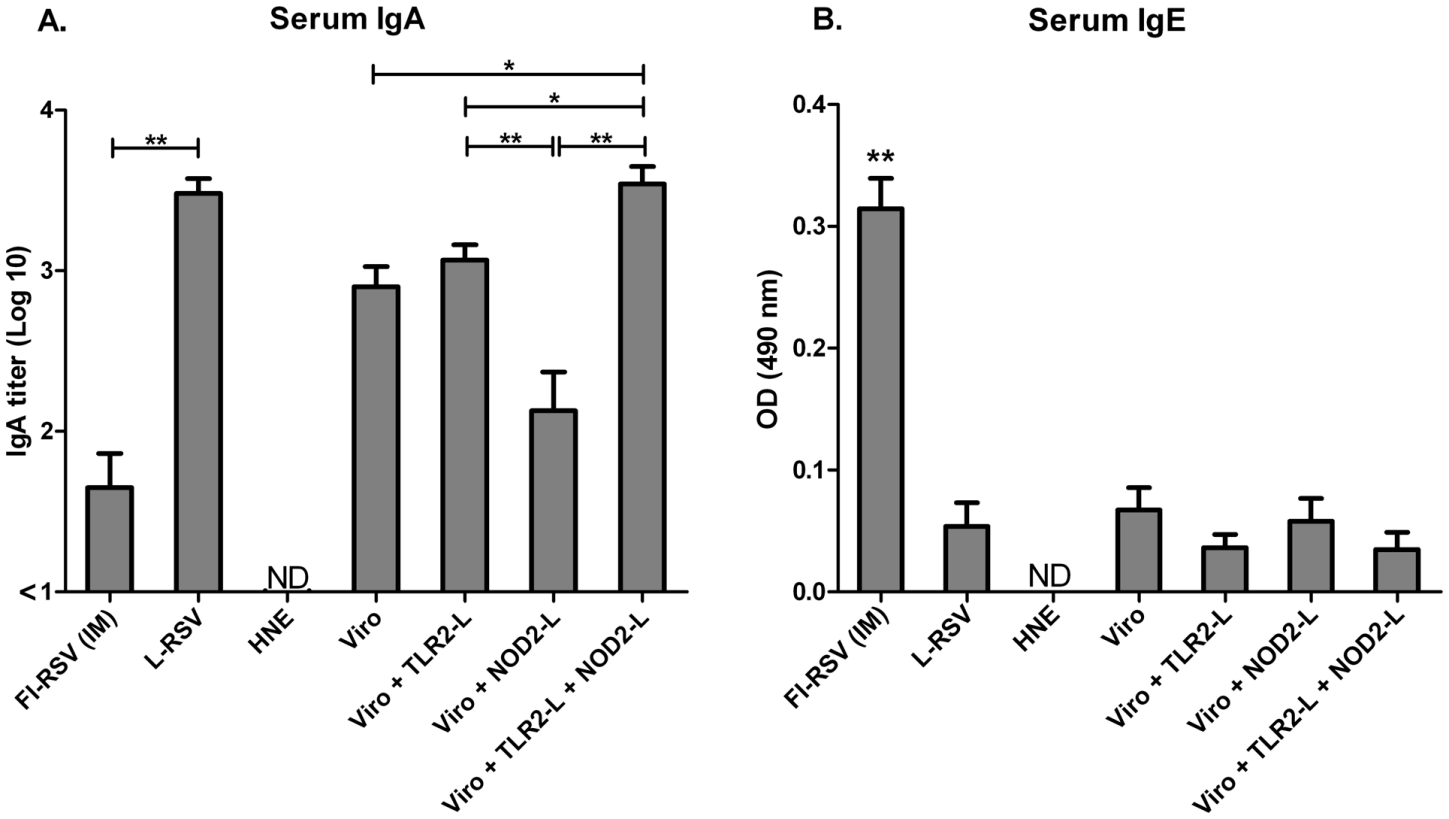
RSV-specific mucosal IgA and IgG antibody responses in nasal washes and BAL after IN immunization of mice. BALB/c mice were immunized IN with RSV virosomal vaccine formulations (5 µg of protein) or HNE. Control mouse groups were either immunized IM with FI-RSV or IN with L-RSV on day 0 and 21. Four days after challenge with live RSV (day 32), RSV-specific IgA responses in nasal washes (A), BAL (C) were determined by ELISA. RSV-specific IgG responses in nasal washes (B) and BAL (D) were also determined. For nasal washes, the data from 6 mice per group is shown and for BAL, data from 3 mice per group is shown. Panels A-D: Bars represent the mean absorbance (490 nm) and standard deviation. The data shown are representative data of at least 3 separate experiments. Data was analyzed by a Mann-Whitney U test and a p-value of ≤ 0.05 was considered to represent a significant difference. * p≤0.05, ** p≤0.01.

To characterize the humoral immune response in more detail, we analyzed RSV-specific serum IgA and IgE antibody levels. Significant levels of RSV-specific serum IgA were induced after immunization with live virus and RSV-virosomes containing Pam_3_CSK_4_, but not after immunization with FI-RSV or virosomes with L18-MDP (Figure 3A). Induction of serum IgE antibodies, a hallmark of atopic responses, was only observed after immunization with FI-RSV (Figure 3B). Thus, RSV-virosomes with incorporated TLR2/NOD2-ligands induce local as well as serum RSV-specific IgA and IgG antibody responses upon mucosal immunization and no IgE antibody responses.

**Figure 4:**
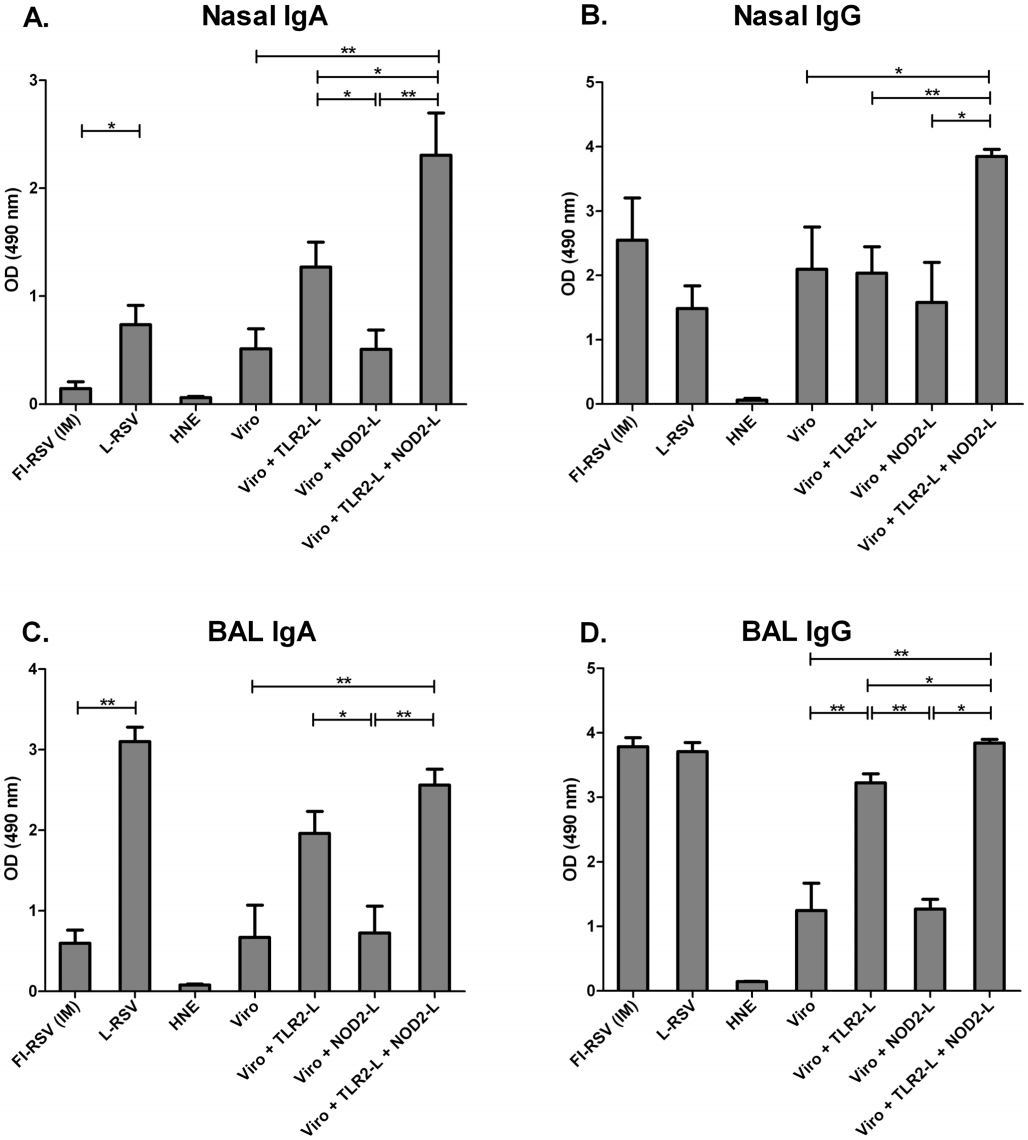
RSV-specific serum IgA and IgE antibody responses. BALB/c mice were immunized IN with RSV-virosomal vaccine formulations (5 µg of protein) or HNE. Control mouse groups were either immunized IM with FI-RSV or IN with L-RSV on day 0 and 21. RSV-specific serum IgA (A) and IgE (B) were determined by ELISA. Data from 6 mice per group is shown. Panel A: Bars represent the geometric mean titer and standard deviation. Panel B: Bars represent the mean absorbance (490 nm) and standard deviation. The data shown are representative data of at least 2 separate experiments. Data was analyzed by a Mann-Whitney U test and a p-value of ≤0.05 was considered to represent a significant difference. * p≤0.05, ** p≤0.01.

In order to determine mucosal immune responses, nasal washes and BAL samples were taken upon sacrifice of challenged animals for analysis of IgA and IgG antibody levels. We found a significant induction of nasal and BAL RSV-specific IgA antibodies in mice immunized IN with RSV virosomes with incorporated Pam_3_CSK_4_ or live virus (Figure 4A,C). Incorporation of L18-MDP in the Pam_3_CSK_4_-virosomes further boosted mucosal IgA antibody responses (Figure 4A,C). We also determined IgG antibody levels in nasal washes and BAL. Nasal RSV-specific IgG was observed in all immunized groups, but the highest levels were seen in the groups immunized IN with virosomes adjuvanted with both ligands (Figure 4B). Immunization with adjuvanted virosomes induced BAL IgG to similar levels as FI-RSV or live virus immunization. Again, additional incorporation of L18-MDP in Pam_3_CSK_4_-adjuvanted virosomes further boosted IgG levels (Figure 4D).

### RSV-specific cell-mediated immune responses

**Figure 5:**
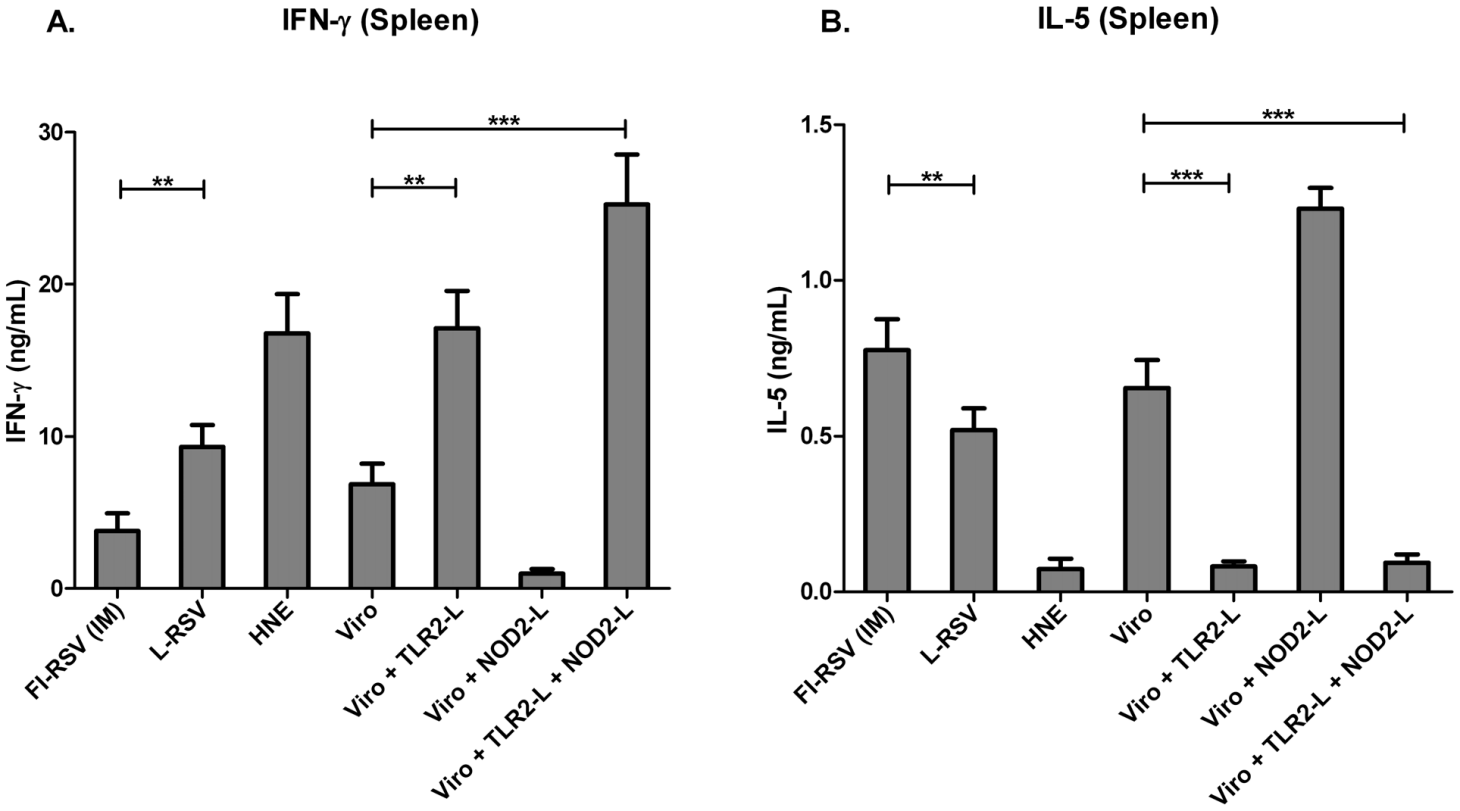
*Ex vivo* cytokine production by splenocytes in response to stimulation with RSV. IFN-γ (Panel A) and IL-5 (Panel B) production in splenocyte cultures after stimulation with inactivated RSV was determined by cytokine ELISA. Cytokines in the culture supernatants were assayed after 3 days of culturing. Data from 6 mice per group is shown. Bars and error bars represent means ± SD. The data shown are representative of at least 3 separate experiments. Data was analyzed by a Mann-Whitney U test and a p-value of ≤ 0.05 was considered to represent a significant difference. * p≤0.05, ** p≤0.01, *** p≤0.001.

As excess Th2-skewed T cell responses may contribute to ERD, we investigated whether the RSV-specific T cell responses had Th1-/or Th2-skewed phenotypes. To this end, we analyzed IFN-γ and IL-5 levels in *ex vivo* RSV-restimulated splenocytes from immunized mice that were challenged by IN inoculation of live virus, one week after the booster immunization. Restimulated splenocytes from non-immune mice that received HNE buffer only produced considerable levels of IFN-γ (Figure 5), which may be explained by activation of innate immunity (i.e. NK cell activation) as a result of administration of a high dose of challenge virus, in the absence of specific immunity. Incorporation of Pam_3_CSK_4_in virosomes induced Th1-skewing: it significantly increased IFN-γ responses while IL-5 responses were significantly reduced (Figure 5). Clear-cut Th2-skewed responses, indicated by high IL-5 but low IFN-γ responses, were induced by FI-RSV and by L18-MDP-adjuvanted RSV virosomes (Figure 5). Incorporation of L18-MDP in Pam_3_CSK_4_-adjuvanted virosomes did not lead to Th2-skewing, but appeared to further increase the Th1-skewing by enhancing IFN-γ secretion (Figure 5). This, however, did not reach a statistically significant difference. Thus, RSV virosomes supplemented with TLR2/NOD2 ligands efficiently prime for safe Th1-phenotype responses upon mucosal immunization in mice.

### Protection from live RSV challenge

**Figure 6:**
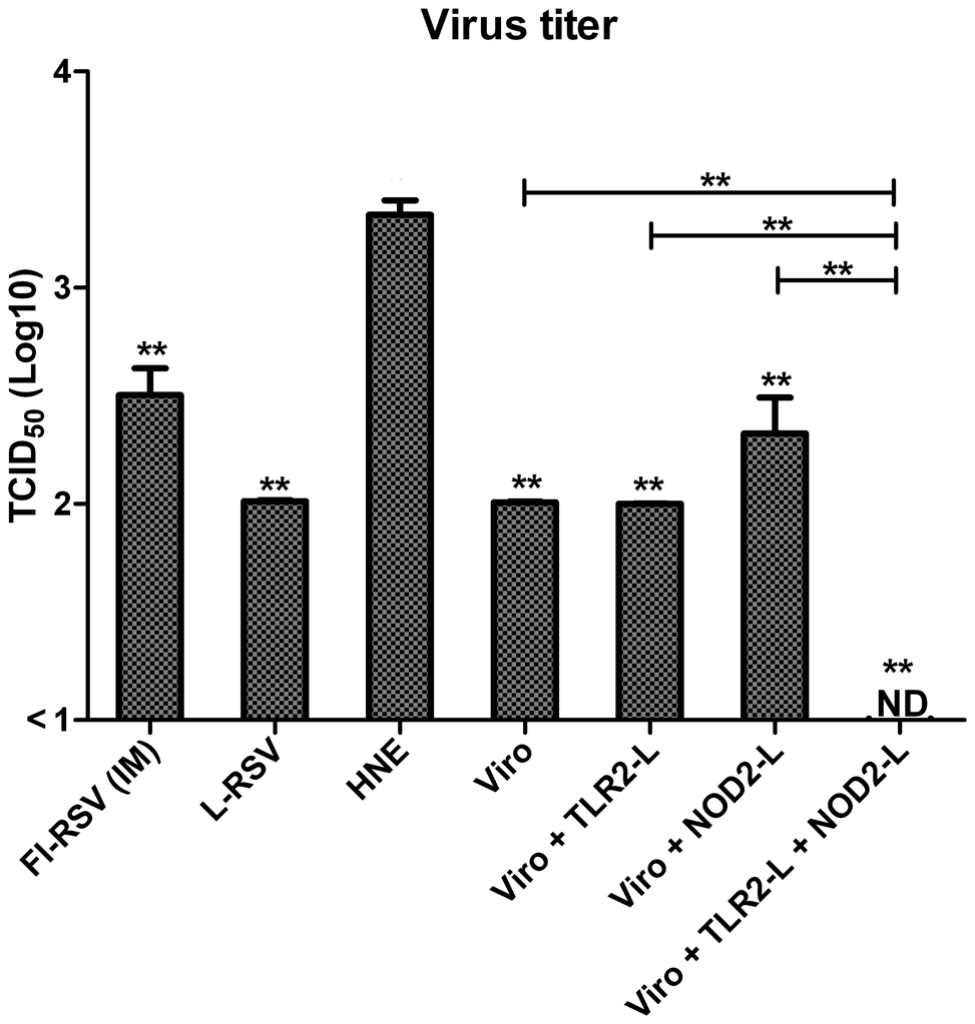
Protection of mice from challenge with live RSV. BALB/c mice were immunized IN with RSV-virosomal vaccine formulations (5 µg of protein) or HNE. Control mouse groups were either immunized IM with FI-RSV or IN with L-RSV on day 0 and 21. Mice were challenged with live-RSV on day 28 and four days after challenge (day 32), lung viral titers were determined. Data from 6 mice per group is shown. Viral titers are expressed as TCID_50_. Bars and error bars represent means ± SD. The data shown are representative of at least 2 separate experiments. Data was analyzed by a Mann-Whitney U test and a p-value of ≤ 0.05 was considered to represent a significant difference. Asterisks indicate groups that had significantly lower viral titers compared to titers in the non-immune HNE group. Horizontal lines compares differences in titers in different immunized groups. * p≤0.05, ** p≤0.01.

In addition to immune parameters, protection against viral challenge was investigated. For this, immunized mice were challenged and four days later, lung viral titers were measured. All immunized mice showed significantly reduced viral titers compared to viral titers seen in non-immunized mice (Figure 6). Only mice immunized with RSV-virosomes with both ligands incorporated had undetectable viral titers (Figure 6).

### Lung pathology

**Figure 7:**
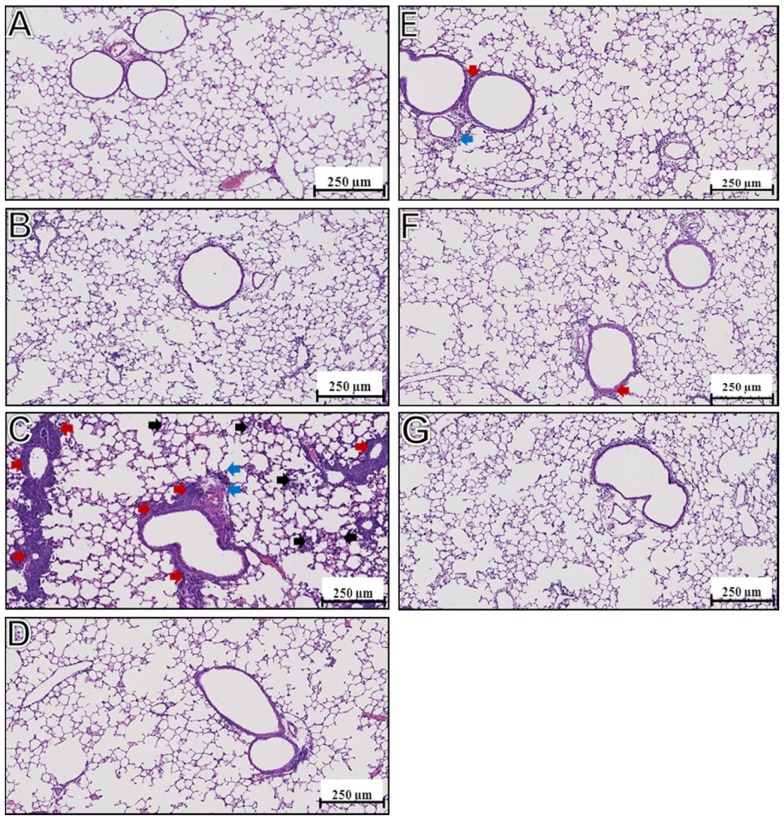
Immunopathology after challenge. BALB/c mice were immunized as described above. One week after the booster immunization, mice were challenged with live RSV (1*10^6^ TCID50). Four days after challenge, one lobe of lung was harvested, sliced and stained with H&E for pathology analysis using light microscopy. Panels are representative pictures of the lungs of mice immunized with (A) HNE, (B) L-RSV, (C) FI-RSV, (D) RSV virosomes, (E) RSV virosomes + TLR2-L, (F) RSV virosomes + NOD2-L and (G) RSV virosomes + TLR2-L + NOD2-L immunized mice. Black arrows indicate alveolitis, red arrows indicate peribronchiolitis and blue arrows indicate perivasculitis.

To examine possible occurrence of ERD upon challenge of immunized mice, lungs were collected four days post-challenge virus and lung slices were examined. Mice immunized with FI-RSV showed clear signs of ERD, i.e. alveolitis and infiltration of cells in peribronchial and perivascular regions (Figure 7C), while non-immunized mice or mice immunized with live virus did not show any signs of ERD (Figure 7A, B). Lungs from mice immunized with non-adjuvanted virosomes, or virosomes with Pam_3_CSK_4_ and/or L18-MDP, did not show signs of ERD either (Figure 7D-G), although some areas with minor infiltration were observed (Figure 7E, F). Thus, unlike IM injection with FI-RSV, IN immunization with (adjuvanted) RSV virosomes does not prime for ERD.

## Discussion

Mucosal delivery of vaccines has been explored as a non-invasive and highly acceptable route of administration and can induce mucosal antibody responses, in addition to systemic antibody responses. Since RSV enters through the respiratory mucosal site, mucosal immunity at these sites would contribute to prevention of infection [26], [27]. However, non-replicating virus vaccines administered through the mucosal route generally induce poor immune responses. This poor immunogenicity may, however, be overcome by co-administeration of mucosal adjuvants with the vaccine [13], [28]–[30]. TLR2-ligands, like MALP-2 (macrophage-activating lipopeptide-2) and zymosan, for example, have been reported to have good mucosal immunoadjuvant properties [16], [17]. Here, we show that the TLR2-ligand Pam_3_CSK_4_, incorporated in RSV virosomes, also has mucosal immuno-adjuvant properties. No clearcut *in vivo* immunoadjuvant activity was observed when the NOD2 ligand L18-MDP was incorporated in virosomes. However, when it was incorporated in virosomes carrying TLR2-ligand, a further increase in *in vivo* antibody responses and Th1-skewing was observed. This points to a synergistic activity of the ligands in immunopotentiation, leading to increased RSV-specific immunity upon IN administration of the virosomal RSV vaccine. From the above data we conclude that Pam_3_CSK_4_, alone or in combination with L18-MDP, shows promise for use as a mucosal adjuvant in a non-replicating virosomal RSV vaccine.

We have previously shown that mucosal immunization with inactivated RSV, supplemented with TLR9 (CpG DNA) and NOD2 (L18-MDP) ligands, is an effective approach for induction of RSV-specific antibodies and Th1-skewed T cell responses [8]. In this study, we investigated the virosome platform as a candidate RSVvaccine, and chose to include the TLR2-ligand Pam_3_CSK_4_, alone or together with L18-MDP. Pam_3_CSK_4_ is a synthetic triacylated lipopeptide that, unlike CpG DNA for example, readily associates with virosomes by partitioning into the virosomal membrane during the reconstitution process (Figure 1) [11]. When incorporated in RSVvirosomes, Pam_3_CSK_4_ enhances RSV-specific serum IgG and Th1 responses upon intramuscular immunization [11]. One way by which it potentiates immune responses is through induction of proinflammatory cytokines, which is initiated after binding to a heterodimeric TLR2/1 receptor and engagement of the MyD88-mediated signaling pathway [31]. A recent study showed that Pam_3_CSK_4_ not only upregulates pro-inflammatory genes, but also genes involved in leukocyte transendothelial migration at the site of vaccine administration [32]. Another possible factor contributing to the adjuvant activity of Pam_3_CSK_4_ could be its cationic nature. This property has been shown to enhance binding and uptake of RSV viral particles by target cells [33]. In a similar fashion, it could enhance binding and uptake of RSV virosomes that contain Pam_3_CSK_4_ by, for example, antigen-presenting cells. Besides this, a number of other activities of TLR2 ligands have been described that could contribute to enhancement of mucosal responses. These include the induction of increased antigen uptake by M cells [34], [35], induction of T-cell-independent B-cell activation and maturation leading to enhanced antibody secretion [16], and enhancement of IgA secretion by B cells [18]. Thus, TLR2-ligands, including Pam_3_CSK_4,_ seem highly suited for immunopotentiation of vaccine-induced systemic and mucosal immune responses upon mucosal administration.

Although the NOD2 ligand L18-MDP has been reported to have mucosal immunoadjuvant activity [19], we observed no enhancement of mucosal antibody responses by virosome-incorporated L18-MDP upon IN immunization of mice. We did, however, observe a strong Th2- skewing by L18-MDP (Figure 5), a feature which has been described before [36]. Both FI-RSV and L18-MDP-adjuvanted virosomes induced Th2-skewed T cell responses, but the latter did not prime for ERD (Figure 7F). Notably, in contrast to FI-RSV, L18-MDP-adjuvanted virosomes do not induce RSV-specific IgE antibodies (Figure 4B), despite the strongly Th2-skewed responses. IgE is an important mediator of hypersensitivity responses including ERD and this may be why mice immunized with L18-MDPvirosomes did not show ERD. The lack of IgE induction may be explained by the active suppression of IgE responses by MDP or its derivatives upon mucosal administration [37]. Thus our data suggest that, on their own, Th2 responses do not readily cause ERD in mice but when associated with RSV-IgE responses contribute to ERD.

L18-MDP did not display Th2-skewing properties when combined with TLR-ligands, such as CpG DNA [8] or Pam_3_CSK_4_ (Figure 5). Rather, L18-MDP enhances the TLR-ligand-mediated activation leading to more pronounced RSV-specific IFN-γ secretion by splenocytes and significantly increased mucosal antibody responses. These data are in line with other studies showing that ligands for NOD-like receptors (NLR), such as NOD2, enhance TLR-ligand-induced activation. For example, the TLR-ligand induced activation, proliferation and survival of B cells was further enhanced by addition of NOD-ligands [23]. Also, TLR-ligand induced (Th1-skewing) cytokines in dendritic cells are further enhanced by supplementing with NOD1/NOD2-ligands [21]. Thus, RSV-specific serum and mucosal antibody responses and Th1 responses boosted by Pam_3_CSK_4_can further be increased by addition of the NOD2-ligand L18-MDP.

As the respiratory tract is the port of entry for RSV, mucosal antibodies in the respiratory tract could significantly contribute to protection. It is likely that mucosal antibodies are important in protection of the upper respiratory tract, while serum antibodies mainly protect the lungs, as has previously been demonstrated for influenza infection [38]. In support of this notion, studies on RSV infection in adult humans and the elderly have shown that nasal antibodies are a better correlate of protection against RSV infection than serum antibodies [39]. On the other hand, high RSV-specific serum IgG has been shown to correlate with reduced disease severity upon RSV infection [40], which points to a role of serum IgG in protection of the lungs. A contribution of the adjuvants on induction of protective immunity is possible, for example through activation of innate immunity. It should be noted however that the RSV-F protein, besides being a major vaccine antigen, is also a TLR4 and TLR2 ligand [41], [42], and thus also has the capacity to activate the innate immunity. To underline the antigen-specific component in the protection afforded by an IN RSV vaccine, we previously found that RSV-specific IgG antibodies induced by IN immunization showed a clear negative correlation with lung viral titers (Spearman r −0.5965, p = 0.0003; [8]). This suggests that serum antibodies are the main contributors to protection of the lower respiratory tract in mice. In line with these data, we earlier found that intramuscular (IM) injection of RSV virosomes adjuvanted with a TLR4 ligand (i.e. MPLA) induced RSV-specific serum IgG capable of protecting the lungs of mice [12]. Like in the present study, that explored the IN route, incorporation of a TLR ligand in IM-injected virosomes similarly enhanced serum IgG levels, IgG2a antibody levels and protection [12]. However, while IN-administered adjuvanted virosomes induced mucosal antibodies, IM-injected adjuvanted virosomes did not (unpublished results).

In conclusion, IN-immunization with RSV-virosomes with incorporated Pam_3_CSK_4_ alone, or combined with the NOD2-ligand L18-MDP is a promising strategy to induce RSV-specific immunity that includes serum and mucosal antibody responses and safe Th1-skewed cellular immune responses, without priming for enhanced disease.
